# Abnormal bodily experiences detected by Abnormal Bodily Phenomena questionnaire are more frequent and severe in schizophrenia than in bipolar disorder with psychotic features

**DOI:** 10.1192/j.eurpsy.2020.49

**Published:** 2020-05-14

**Authors:** Giovanni Stanghellini, Davide Palumbo, Massimo Ballerini, Armida Mucci, Francesco Catapano, Giulia Maria Giordano, Silvana Galderisi

**Affiliations:** 1 Department of Psychological, Humanistic and Territorial Sciences, G. D’Annunzio University, Chieti, Italy; 2 D. Portales University, Santiago, Chile; 3 Department of Psychiatry, University of Campania “Luigi Vanvitelli”, Naples, Italy; 4 Department of Mental Health, Florence, Italy

**Keywords:** Abnormal bodily phenomena, assessment, negative symptoms, phenomenology, psychotic symptoms

## Abstract

**Background:**

Patients with schizophrenia display experiential anomalies in their feelings and cognitions arising in the domain of their lived body. These abnormal bodily phenomena (ABP) are not part of diagnostic criteria for schizophrenia. One of the reasons is the difficulty to assess specific ABP for schizophrenia spectrum disorders. The present study aimed to explore the presence in patients with schizophrenia of specific ABP.

**Methods:**

We used a semistructured interview—the Abnormal Bodily Phenomena questionnaire (ABPq), an instrument devised to detect and measure ABP specific to patients with schizophrenia. Fifty-one outpatients affected by schizophrenia and 28 euthymic outpatients affected by bipolar disorder type I with psychotic features (BD-pf-e) were recruited. Before assessing the specificity for schizophrenia of the observed ABP, we tested the internal consistency and the convergent validity of the ABPq in patients with schizophrenia. Specificity was assessed by examining potential differences in ABPq among the patients with schizophrenia in remission (SCZ-r) and BD-pf-e.

**Results:**

The ABPq shows strong internal consistency and convergent validity. As to the specificity, ABP measured by ABPq were more frequent and severe in SCZ-r than in BD-pf-e. In particular, all ABPq dimensions, except “Coherence,” had at least mild severity in over 50% of SCZ-r, while dimensions with at least mild severity were observed in 5–10% of the BD-pf-e.

**Conclusions:**

These findings can contribute to establish more precise phenomenal boundaries between schizophrenia and bipolar disorder, to explore the borders between nonpsychotic and psychotic forms of ABP, between ABP and negative and disorganized symptoms, and to enlighten core aspects of schizophrenia.

## Introduction

Notwithstanding theoretical and scientific advances in the study of schizophrenia, the nuclear aspects of the syndrome are still debated. Current nosography is not based on a strong explanatory paradigm of the disorder, and a coherent framework for the data provided by scientific research is still lacking. Boundaries between schizophrenia and other psychoses appear arbitrary, and no specific pathophysiology or biomarker has been identified so far [1,2]. An oversimplification of psychopathology might have contributed to this state of affairs. As observed by Maj, the operational diagnostic criteria of the Diagnostic and Statistical Manual of Mental Disorders (DSM)-IV and DSM-5 mainly clarify what schizophrenia is not (e.g., nonorganic, nonaffective, etc.), rather than defining what it is [3]. Parnas emphasized that the issue raised by Maj does not involve a commitment to realism about natural kinds, but is prompted by clinical experience and based on the assumption that schizophrenia has a nuclear aspect that gives it a certain typicality [4]. In this context, the efforts of several researchers focused on defining the nuclear aspects of the syndrome, that is, trait features that reflect the phenomenological structure of the syndrome, instead of fluctuating state phenomena such as psychotic symptoms [5–8]. Recently, emphasis has been placed on several well-known concepts of classical psychopathology that have been neglected by the current mainstream nosography; one of them is the construct of abnormal bodily phenomena (ABP).

### Abnormal bodily phenomena

ABP have been observed in patients with psychosis, and particularly in patients with schizophrenia, since the early categorizations of the syndrome [9]. ABP refer to different types of symptoms: disturbed coenesthesia, kinesthetic hallucinations, and disruptions of body structure and boundaries [10]. From a phenomenological perspective, ABP underlie subjective experiential anomalies in the feelings, sensations, perceptions, and cognitions that emerge in the domain of the lived body [11]. Phenomenology postulates a distinction between the lived body (*Leib*) and the physical body (*Koerper*). The first is the body experienced “from within”: the subjective and immediate experience of one’s body, that is, the center of the most primitive form of self-awareness. The second is the body experienced “from without,” from a third-person perspective, such as the body investigated by natural sciences [12]. The embodied form of self-awareness is regarded as the basis of the differentiation between Self and Other, between “oneself” and the “perceived objects” [13]. ABP are therefore considered an alteration of the basic and prereflective form of the Self and, according to some authors, a fundamental feature of schizophrenia [13–16]. ABP are included among the basic symptoms described by Huber [17]—perhaps the first detailed description of ABP—for example, slight (but source of discomfort) disturbances of drives, affections, perception, proprioception, motility, and vegetative function [18]. On this description are grounded the Bonn Scale for the Assessment of Basic Symptoms (BSABS, scale D) [19], in which a list of ABP is included, and the Schizophrenia Proneness Instrument, Adult version subscale E “Body Perception Disturbances” (SPI-A, scale E) [20]. Research on ABP in patients with schizophrenia using the BSABS showed that a subset of bodily basic symptoms are core predictors of the transformation of the lived space in schizophrenia [21] and central features of schizophrenia spectrum even in its subclinical tails [22]. The Examination of Anomalous Self-Experience (EASE, scale C) [23] and the Perceptual Aberration Scale (PER) [24] also explore ABP. A significant number of studies have shown that these phenomena are present both in patients with schizophrenia and in those at risk [10,25–27]. Nevertheless, ABP are not currently part of mainstream diagnostic criteria for schizophrenia and are rarely assessed, probably due to (a) the lack of reliable methods for their evaluation and (b) the difficulty in defining specific ABP in schizophrenia.

ABP included in BSABS could not discriminate schizophrenia from other psychiatric disorders [28] and displayed unsatisfactory psychosis transition accuracy in the Cologne Early Recognition study [29]. In a 7-year follow-up study, the EASE showed to be able to discriminate the schizophrenic spectrum psychoses from nonschizophrenic psychoses [30]. However, among the different psychopathological dimensions investigated by EASE, the “Bodily Experiences” dimension resulted to be the least discriminative. In conclusion, ABP included in the PER were documented in stable patients with schizophrenia, but they did not show a good accuracy-rating in the prediction of schizophrenia [31]. We suggest that these researches on ABP are very promising although the inconsistencies and the limited diagnostic utility demonstrated by these assessment tools may arise from a too broad definition of ABP, probably including many unspecific anomalies of bodily experience.

### Study aims

The present study aimed to explore the prevalence of ABP in patients with schizophrenia and in those with bipolar disorder type I with psychotic features in a euthymic phase, and to demonstrate its specificity for schizophrenia. We used a semistructured interview—the Abnormal Bodily Phenomena questionnaire (ABPq) [11,32], an instrument devised to measure a set of bodily complaints, developed from qualitative research in the domain of abnormal bodily sensations in patients with first-episode and chronic schizophrenia [11,33].

## Methods

### Study participants

Fifty-one outpatients affected by schizophrenia (SCZ-p) and 28 euthymic outpatients (e) with bipolar disorder type I (BD) with psychotic features (pf) who experienced one or more recent episodes of depression or mania with psychotic features (BD-pf-e) were recruited from those consecutively seen from January 2016 to May 2017 at the outpatient unit for psychotic or mood disorders of the Department of Psychiatry of the University of Campania “Luigi Vanvitelli,” who accepted to participate in the study. Inclusion criteria were a diagnosis of schizophrenia or bipolar disorder type I with psychotic features, according to DSM-IV criteria, confirmed by the Structured Clinical Interview for DSM-IV–Patient Version (SCID-I/P. Exclusion criteria were: (a) neurological diseases; (b) history of alcoholism or substance abuse; (c) inability to provide informed consent; (d) mild, moderate, or severe intellectual disability; and (e) changes in antipsychotic or mood-stabilizer medications or hospitalization within 3 months prior to the inclusion in the study.

The study was approved by the Ethics Committee of the University Hospital of the University of Campania Luigi Vanvitelli. All patients signed a written informed consent before undergoing study procedures.

### Instruments

The following scales were administered to the study participants:

1. The ABPq explores the lived experience of the body and investigates the presence of ABP in patients with schizophrenia [11,32]. The phenomena include five dimensions: Demarcation, that is, experiences of violation of bodily boundaries (it includes intrusions of external entities into one’s body or externalization of bodily parts); Vitality, that is, experiences of one’s body or its parts as inert/lifeless things (it includes morbid objectivization and devitalization); Coherence, that is, experiences of decomposition of the internal structure or *Gestalt* of one’s body; Identity, that is, experiences of transformations of one’s body and dysmorphic phenomena; and Activity, that is, unpleasant or painful feelings in one’s body (it includes dysesthesic paroxysms and pain-like phenomena). Severity is scored on a scale from 1 to 7 (higher scores correspond to greater severity) by taking into account frequency, intensity of subjective arousal or distress impairment and capacity to cope. The interview takes from 30 to 60 min.
2. The Positive and Negative Syndrome Scale (PANSS) is a 30-item clinical scale which evaluates general psychopathology, positive, and negative symptoms [34]. Every item is rated on a 7-point symptom severity scale, ranking from 1 (absent) to 7 (extremely severe). In this study, ratings on PANSS items were summed to calculate two dimensions of schizophrenia symptomatology, according to the method proposed by Wallwork et al. [35]: the positive dimension, calculated by summing the items delusions, hallucinatory behavior, grandiosity, and unusual thought content, and the disorganization dimension, calculated by summing the items conceptual disorganization, difficulty in abstract thinking, and poor attention.
3. The Brief Negative Symptoms Scale (BNSS) was administered to evaluate the severity of the negative symptoms; it consists of 13 items organized in six subscales: anhedonia, distress, asociality, avolition, blunted affect, and alogia [36]. All the items are rated on a 7-point scale (0–6), with total scores ranging from 0 to 78. The highest score is associated with the greatest severity of symptoms. The total score of the BNSS is calculated by summing the ratings from all the items except for the item “distress.” The Italian version of the scale was validated as part of the Italian Network for Research on Psychoses activities [37].
4. The SPI-A E was administered to assess coenesthopathies [20]. It is a semistructured interview derived from the BSABS. The SPI-A E investigates six different dimensions of the phenomenon: (a) abnormal sensations of numbness and muscular tension; (b) abnormal delimited painful sensations; (c) abnormal sensations moving along the body; (d) atypical sensations, impression of being electrocuted; (e) feeling of movement, tension, or pressure inside or on the body surface; and (f) bodily sensations of shrinkage, tightening, constriction, enlargement, or expansion. The range of severity is between 0 (absent) and 6 (extreme).

### Training of evaluators and assessment of inter-rater reliability

The assessment was conducted by three residents in Psychiatry properly trained for the administration of the instruments. Both for the PANSS, BNSS, and SPI-A, the three evaluators achieved a certificated training. The training for the administration of the ABPq was conducted by one of the authors of the instrument, and an excellent agreement was observed among raters (intraclass correlation coefficient ranging from 0.77 and 0.98). Further information on the procedure of the training and inter-rater reliability analysis can be found in Stanghellini et al. [32].

### Statistical analysis

All statistical analyses described below were conducted using IBM SPSS Statistics Version 22. The significance level for all statistical comparisons was set at *p* < 0.05.

Before assessing the specificity for schizophrenia of the observed ABP, we tested the internal consistency and the convergent validity of the ABPq.

#### Internal consistency

The ABPq internal consistency was evaluated using Cronbach’s alpha in the patients affected by schizophrenia (SCZ) sample.

#### Convergent validity

In the SCZ sample, ABPq convergent validity was assessed by examining its correlations (both total and dimension scores) with the PANSS positive and disorganization dimensions, and the SPI-A E coenesthopathies. A Bonferroni correction for multiple comparisons was applied to control for type 1 error.

#### Specificity

The specificity was analyzed by comparing the frequency and severity of the ABPq items between subjects with schizophrenia showing remission of positive symptoms, according to the severity criteria proposed by Andreasen et al. [38], and euthymic subjects with bipolar disorder type I with psychotic features (who experienced one or more recent episodes of depression or mania with psychotic features). Positive symptom symptomatic remission was characterized as a score <4 (i.e., absent to mild) on the following PANSS items: “P1. Delusions,” “P2. Conceptual disorganization,” “P3. Hallucinatory behavior,” “P6. Suspiciousness/persecution,” and “G9. Unusual thought content.”

A one-way analysis of variance (ANOVA) was used to test differences between remitted patients with schizophrenia (SCZ-r) and BD-pf-e with respect to age, education, and duration of illness. The two clinical populations were also compared for sex distribution by the *χ* ^2^ test.

In order to assess differences in the frequency of symptoms, the number of symptoms of at least mild severity (i.e., with a score ≥3) was computed in both groups. Subsequently, the data obtained were compared by the *χ* ^2^ test.

Differences in symptom severity between the two groups were tested using a multivariate analysis of variance (MANOVA), with dimensions of the scale (Demarcation, Vitality, Coherence, Identity, and Activity) as within-subject factors and diagnosis as between-subject factor (SCZ-r and BD-pf-e). Follow-up univariate ANOVAs for investigation of simple effects were carried out only when significant group main effects or interactions were found in the MANOVA.

## Results

### Socio-demographic and clinical characteristics

The SCZ sample was composed by 51 subjects, 33 (64.7%) men, with a mean age of 40.33 (standard deviation [SD] ± 10.82) years, mean education of 13.57 (SD ± 3.05) years, and mean illness duration of 17.8 (SD ± 9.96) years. Twenty-six patients with schizophrenia had a symptomatic remission of the positive symptoms (SCZ-r).

No statistically significant difference was found between the SCZ-r group and BD-pf-e for gender distribution (*χ* ^2^ = 0.28; *p* = 0.60), age (*F* = 2.04; *p* = 0.16), education (*F* = 0.67; *p* = 0.41), and duration of illness (*F* = 0.56; *p* = 0.46). The socio-demographic and clinical characteristics of the study groups are illustrated in Table 1.

**Table 1.** Characteristics of the study groups.

	SCZ (*n* = 51)	SCZ-r (*n* = 26)	BD-pf-e (*n* = 28)
Men (%)	64.7	50	57.14
Age (mean years ± SD)	40.33 ± 10.82	37.19 ± 11.63	41.29 ± 9.39
Education (mean years ± SD)	13.57 ± 3.05	14.19 ± 3.02	13.25 ± 5.07
Illness duration (mean years ± SD)	17.8 ± 9.96	14.15 ± 9.99	16.29 ± 10.9

Abbreviations: BD-pf-e, subjects affected by bipolar disorder type I with psychotic features during a euthymic phase; SCZ, subjects affected by schizophrenia; SCZ-r, subjects affected by schizophrenia, in remission.

### Internal consistency

The ABPq internal consistency was evaluated using Cronbach’s alpha in the SCZ sample. Internal consistency for each item was calculated considering the quantitative features (frequency, intensity, impairment, and need for coping). The internal consistencies for ABPq dimensions were calculated using the total score for each item, and the total consistencies for each scale’s dimensions were calculated using the scores for the 16 items. The internal consistency was very high (*α* = 0.893) indicating excellent psychometric properties of ABPq.

### Convergent validity

In the SCZ sample, ABPq convergent validity was assessed by examining its correlations (both total and dimension scores) with the PANSS positive and disorganization dimensions, the BNSS total score and dimensions, and the SPI-A E coenesthopathies. A Bonferroni correction for multiple comparisons was applied to control for type 1 error.

The ABPq total score was significantly correlated with the SPI-A E” (*r* = 0.9, *p* < 0.001). All ABPq dimensions showed a moderate to high correlation with all the items of the SPI-A E, except for the “Vitality” dimension that showed a moderate correlation only with the dimensions “abnormal sensations moving along the body,” “Atypical sensations, impression of being electrocuted,” and “bodily sensations of shrinkage, tightening, constriction, enlargement, or expansion” as illustrated in Table 2.

**Table 2.** Analysis of the correlations of ABPq total scores, ABPq dimensions, and other psychopathological scales in the total sample of patients affected by schizophrenia.

	Demarcation	Vitality	Coherence	Identity	Activity	ABPq total
BNSS anhedonia	0.259	0.359**	0.033	0.151	0.074	0.241
BNSS distress	0.020	0.158	0.008	0.079	−0.127	0.059
BNSS asociality	0.144	0.274	−0.074	−0.017	−0.030	0.091
BNSS avolition	0.043	0.298*	−0.058	0.104	0.001	0.116
BNSS blunted affect	0.0729	0.228	−0.016	0.075	−0.063	0.096
BNSS alogia	0.104	0.220	0.024	0.125	−0.035	0.132
BNSS total score	0.148	0.320*	−0.014	0.107	−0.021	0.160
SPI-A E1	0.348*	−0.032	0.559**	0.524**	0.577**	0.462**
SPI-A E2	0.378**	0.144	0.572**	0.592**	0.854**	0.579**
SPI-A E3	0.889**	0.360**	0.885**	0.752**	0.655**	0.879**
SPI-A E4	0.880**	0.341*	0.754**	0.636**	0.577**	0.796**
SPI-A E5	0.797**	0.242	0.954**	0.820**	0.724**	0.866**
SPI-A E6	0.642**	0.401**	0.821**	0.944**	0.726**	0.879**
SPI-A E total score	0.793**	0.297*	0.906**	0.847**	0.813**	0.896**
PANSS pos	0.554**	0.359**	0.389**	0.293*	0.323*	0.479**
PANSS dis	0.359**	0.353*	0.384**	0.469**	0.389**	0.487**

Abbreviations: ABPq, Abnormal Bodily Phenomena questionnaire; BNSS, Brief Negative Symptoms Scale; SPI-A E, Schizophrenia Proneness Instrument, Adult version subscale “Body Perception Disturbances”; SPI-A E1, SPI-A E “abnormal sensations of numbness and muscular tension”; SPI-A E2, SPI-A E “abnormal delimited painful sensations”; SPI-A E3, abnormal sensations moving along the body; SPI-A E4, SPI-A E “atypical sensations, impression of being electrocuted”; SPI-A E5, SPI-A E “feeling of movement, tension or pressure inside or on the body surface”; SPI-A E6, SPI-A E “bodily sensations of shrinkage, tightening, constriction, enlargement, or expansion”; PANSS pos, Positive and Negative Symptoms Scale, positive dimension; PANSS dis, Positive and Negative Symptoms Scale, disorganization.

* *p* < 0.05.

** *p* < 0.01.

The ABPq total score was also significantly correlated with the two dimensions “Positive symptoms” and “Disorganization” of the PANSS (*r* = 0.48 and *r* = 0.49, *p* < 0.01 and *p* < 0.01, respectively). The PANSS positive dimension and disorganization showed a moderate correlation with all the ABPq dimensions, as shown in Table 2. The ABPq total score had no correlation with negative symptoms (*r* = 0.16; *p* > 0.09); however, the dimension “Vitality” had a moderate positive correlation with the BNSS total score, due to the correlations with the BNSS subscales “Anhedonia” and “Avolition” (Table 2).

### Specificity

#### Frequency of ABPq symptoms

**Figure 1.**
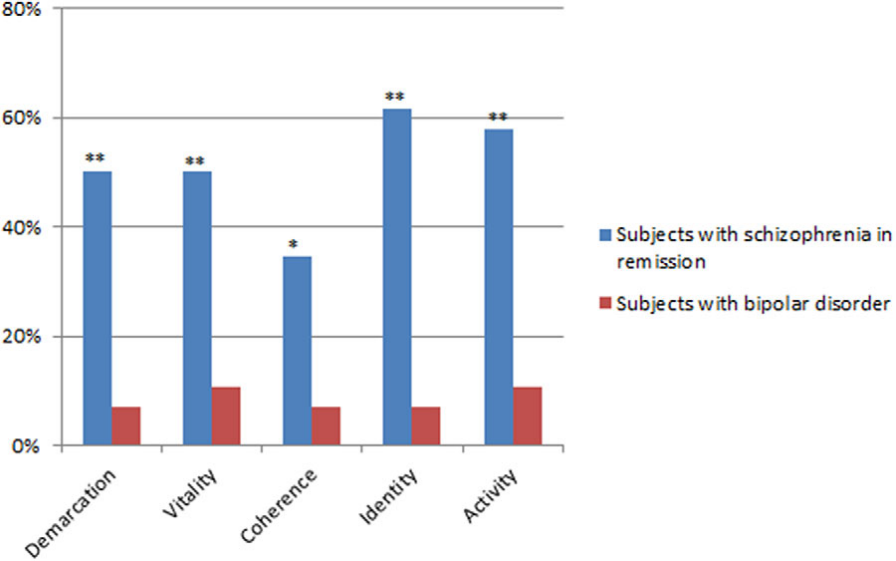
Frequency (%) of ABPq dimensions of at least mild severity (≥3). *p < 0.05; **p < 0.001.

Chi-squared tests comparing the distribution of ABPq dimensions of at least mild severity (≥3) between the SCZr and BD-pf-e group (Figure 1) showed statistically significant difference for all ABPq dimensions. In particular, all dimensions, except “Coherence,” had at least mild severity in over 50% of SCZ-r.

#### Severity of ABPq symptoms

MANOVA showed an interaction group × dimensions (*F* = 9.03, *p* < 0.000001). The mean scores on the ABPq dimensions were significantly higher in SCZ-r than in BD-pf-e, except for the “Vitality” dimension (Table 3).

**Table 3.** Severity of ABPq dimensions in the two patient groups.

	SCZ-r (*n* = 26)		BD-pf-e (*n* = 28)		*p*/ANOVA
	Mean ± SD	Range	Mean ± SD	Range	
Demarcation	4.35 ± 2.48	2–9	2.25 ± 0.70	2–5	0.0001
Vitality	2.92 ± 1.47	2–8	2.39 ± 1.28	2–8	0.16
Coherence	2.35 ± 1.79	1–6	1.18 ± 0.54	1–3	0.01
Identity	5.46 ± 3.51	2–12	2.21 ± 0.68	2–5	0.0001
Activity	4.88 ± 2.63	2–10	2.36 ± 1.06	2–7	0.0001
ABPq global score	19.96 ± 9.81	9–37	10.39 ± 2.54	9–17	0.00001

The table shows the average scores obtained by SCZ-r and BD-pf-e subjects in all ABPq domains (mean ± SD) and the minimum and maximum scores (range) obtained in each group.

Abbreviations: ABPq, Abnormal Bodily Phenomena questionnaire; BD-pf-e, subjects affected by bipolar disorder type I with psychotic features during a euthymic phase; SCZ-r, subjects affected by schizophrenia, in remission; SD, standard deviation.

## Discussion

The ABPq is a semistructured interview showing strong convergent validity and specificity providing clinicians and researchers with a detailed characterization and operationalized definition of ABP, with a structured set of prompts to elicit them, and with a scale to assess their severity.

All the ABPq categories were strongly related to all the items of SPI-A E [27]. This finding is supportive of the convergent validity of ABPq. To the best of our knowledge, there is no evidence that SPI-A E discriminates between patients with schizophrenia and bipolar disorder. Instead, the ABPq proved to be effective in separating SCZ-r from BD-pf-e.

In our study, specificity was studied by matching the SCZ-r with BD-pf-e to identify vulnerability trait-like psychopathological features, that is, features that persist in stable patients after clinical remission of psychotic symptoms. SCZ-r generally display some residual symptomatology, while patients with euthymic bipolar may be asymptomatic. However, the possible difference in severity of residual psychotic symptoms does not seem to explain our results. In fact, ABP represent stable trait-like aspects which might be present during complete remission of psychotic symptoms. As a matter of fact, patients with euthymic bipolar did show ABP phenomena, while they did not have psychotic symptoms. Furthermore, for the “Vitality” ABP the mean severity is the same in both groups. Finally, in the SCZ sample, the correlations of ABP with the PANSS scores for psychotic symptoms are not in the high to strong range, but much lower (all ≤ 0.55) than those expected were the ABP and the expression of the severity of psychotic symptoms. Correlations in the range high to very strong (0.70–0.89) are observed only with the SPI-A scores, which measure similar trait-like phenomena.

The specificity of ABPq is thus supported by the following findings: (a) the severity of ABP measured with ABPq was significantly higher in SCZ-r than in BD-pf-e. (b) ABP were detected with a significantly higher frequency in SCZ-r than in BD-pf-e. Specifically, SCZ-r showed an ABP frequency in about 50% of cases, while BD-pf-e in 10–15% of cases.

The only exception is the “Vitality” ABP which has comparable severity in SCZ-r and BD-pf-e, possibly indicating that this type of ABP represents a vulnerability trait of psychosis in general.

In conclusion, the ABPq, better than other scales, identifies a specific group of ABP which may discriminate patients with schizophrenia from patients with nonschizophrenia-spectrum, thus displaying diagnostic utility. These ABP are well documented in (at least) a subsample of SCZ-r; this suggests that ABPq abnormal bodily phenomena may be considered stable trait-characters and/or mediating vulnerability factors (the latter exacerbated in acute psychosis) since they encompass not-yet-psychotic and full-blown psychotic phenomena (see the following section).

Also, the ABPq (as well as the SPI-A) provides Likert scoring system capable to capture slight differences and degrees of severity of abnormal bodily experiences, whereas other scales (including the EASE as it is usually performed) provide only a dichotomous (present/absent) system of scoring.

### ABPq and psychotic symptoms

The ABPq total score was significantly and robustly correlated with the PANSS positive subscale, and all its constitutive dimensions demonstrated at least mild correlation with the positive PANSS subscale.

The boundaries between nonpsychotic and psychotic ABP are not easy to define [32]: ABP can be considered full-blown psychotic symptoms when they overpass the “as if” modality, or when they result as (cognitive) thematic elaborations of primary experiences [4]. This is the case, for instance, of the transition from abnormal experiences of diminished vitality (e.g., “My head as if fogged up on left, as if it’s not working, as if I’m only thinking on one side“) to delusions of alien control (e.g., “Device implanted into the back of my head to control me”).

ABPq can help discriminate between nonpsychotic and full-blown psychotic ABP since it distinguishes the *experiential features* of the ABP from pseudo-explanatory constructs or *causes* as reported in the patients’ narratives. When patients confine themselves to report ABP using images or metaphors (e.g., “My head as if fogged up on the left”) these phenomena can be diagnosed as *nonpsychotic* ABP, whereas when patients try to explain them in terms of their subjectively supposed causes, (e.g., specific devices used to control one’s body or actions), or using neologistic verbal constructs (e.g., “foggizator” apparatus, i.e., an apparatus to produce “fog” in one’s head), we are in presence of *psychotic* ABP, since psychotic symptoms can be considered a superstructure construed by the patient as a pseudo-explanation of aberrant and disturbing “basic” anomalies of experience.

It may be argued that subtle disturbances of embodiment (e.g., nonpsychotic ABP) may be part of the larger category of self-disorders and that nonpsychotic (or not-yet-psychotic) abnormal bodily experiences may represent the origin of full-blown psychotic symptoms as, for instance, somatic hallucinations and delusions of bodily control. In this sense, ABP may be considered “mediating vulnerability factors.” In every case, we need longitudinal studies to confirm this hypothesis.

### ABPq and negative/disorganized symptoms

The boundaries between ABP and negative symptoms is also controversial. The ABPq dimension “Vitality” (which includes experiences of mechanization and morbid objectivation) was related to the total score of BNSS (*r* = 0.320) and specifically with two dimensions of negative symptoms, anhedonia and avolition, that load on the same construct in factorial analyses of the BNSS [36,37]. Future research should test the hypothesis that ABPq anomalies of vitality may represent a possible root for negative symptomatology.

## Conclusion

The present study demonstrates that the ABP, measured by ABPq, are more frequent and severe in patients with schizophrenia than in those with bipolar disorder type I with psychotic features assessed during a phase of euthymia. These findings can contribute to establish more precise phenomenal boundaries between schizophrenia and bipolar disorder, to explore the borders between nonpsychotic and psychotic forms of ABP, the differences between ABP and negative and disorganized symptoms, and finally to enlighten one of schizophrenia nuclear aspects.

## Data Availability

The data that support the findings of this study are available from the author, A.M., upon reasonable request.
